# The association between population health management tools and clinician burnout in the United States VA primary care patient-centered medical home

**DOI:** 10.1186/s12875-024-02410-8

**Published:** 2024-05-15

**Authors:** Jane Wang, Lucinda Leung, Nicholas Jackson, Michael McClean, Danielle Rose, Martin L. Lee, Susan E. Stockdale

**Affiliations:** 1 Department of Medicine, Stanford University, Stanford, USA; 2 Department of Medicine, David Geffen School of Medicine, UCLA, Los Angeles, USA; 3 Center for the Study of Healthcare Innovation, Implementation & Policy, VA Greater Los Angeles Healthcare System, 16111 Plummer Avenue, North Hills, CA 91343 USA; 4 Department of Biostatistics, UCLA Fielding School of Public Health, Los Angeles, USA; 5 Department of Psychiatry and Biobehavioral Sciences, UCLA, Los Angeles, USA

**Keywords:** Population health management tools, Burnout, Information technology burden, Panel management

## Abstract

**Background:**

Technological burden and medical complexity are significant drivers of clinician burnout. Electronic health record(EHR)-based population health management tools can be used to identify high-risk patient populations and implement prophylactic health practices. Their impact on clinician burnout, however, is not well understood. Our objective was to assess the relationship between ratings of EHR-based population health management tools and clinician burnout.

**Methods:**

We conducted cross-sectional analyses of 2018 national Veterans Health Administration(VA) primary care personnel survey, administered as an online survey to all VA primary care personnel (*n* = 4257, response rate = 17.7%), using bivariate and multivariate logistic regressions. Our analytical sample included providers (medical doctors, nurse practitioners, physicians’ assistants) and nurses (registered nurses, licensed practical nurses). The outcomes included two items measuring high burnout. Primary predictors included importance ratings of 10 population health management tools (eg. VA risk prediction algorithm, recent hospitalizations and emergency department visits, etc.).

**Results:**

High ratings of 9 tools were associated with lower odds of high burnout, independent of covariates including VA tenure, team role, gender, ethnicity, staffing, and training. For example, clinicians who rated the risk prediction algorithm as important were less likely to report high burnout levels than those who did not use or did not know about the tool (OR 0.73; CI 0.61-0.87), and they were less likely to report frequent burnout (once per week or more) (OR 0.71; CI 0.60-0.84).

**Conclusions:**

Burned-out clinicians may not consider the EHR-based tools important and may not be using them to perform care management. Tools that create additional technological burden may need adaptation to become more accessible, more intuitive, and less burdensome to use. Finding ways to improve the use of tools that streamline the work of population health management and/or result in less workload due to patients with poorly managed chronic conditions may alleviate burnout. More research is needed to understand the causal directional of the association between burnout and ratings of population health management tools.

**Supplementary Information:**

The online version contains supplementary material available at 10.1186/s12875-024-02410-8.

## Introduction

Workplace burnout affects approximately half of all physicians [1] and 35–45% of nurses [2] in the United States. It is associated with an increased risk of major medical errors [3], worse quality of care [4], and decreased patient satisfaction [4]. It has been calculated to drive $5 billion per year in lost clinical productivity and physician turnover in the United States alone [5], and has resulted in the erosion of clinician health and well-being, with 14% of physicians reporting suicidal thoughts in 2019 [1], and up to 51% of nurses reporting suicidal thoughts during the COVID-19 pandemic [6]. Though burnout has affected practitioners across a wide spectrum of clinical specialties, it has particularly impacted primary care clinicians, who are more likely than other medical providers to experience workplace emotional exhaustion [7]. Some evidence suggests that burnout is higher among primary care medical doctors (MDs) as compared with nurse practitioners or physician’s assistants [8]. Clinicians who carry larger proportions of patients with high care coordination needs are more likely to suffer from burnout [9], perhaps due to the potential impact of population health management on workload [10]. Patients with multiple morbidities are more likely to require preventive care, ongoing medical management, and care coordination [11–13].

Population health management is a key component of patient centered medical homes (PCMH) and is usually performed by primary care providers and nurses. It is defined as the improvement of a population’s health through defined models of care coordination and patient engagement processes [14]. The goal of population health management is to implement prophylactic health practices to prevent onset or progression of disease while simultaneously reducing health care costs from complex medical hospitalizations or procedures [15]. Some healthcare systems have introduced population health management tools (PHMTs), typically embedded within the electronic healthcare record, to facilitate medical care by PCMH teams. Several commercial EHR vendors offer PHMTs as part of their packages, including Epic [16], Cerner [17], Meditech [18], Athenahealth [19], and NextGen Healthcare [20]. Examples of such tools include data integration, analytics, and visualization [21]; patient panel dashboards [22–26];artificial intelligence algorithms [27]; health maintenance reminders and best practice alerts [28].

These tools are primarily intended to improve care quality and reduce downstream costs of more expensive care for patients with uncontrolled chronic conditions. They may also streamline the work of population health management by helping clinicians identify and manage high-risk patients, thus reducing the workload associated with this task. Many population health management tools, however, generate increased technological burden through more electronic health record (EHR) alerts and requisite dashboard views. A growing body of literature has demonstrated a strong association between technological burden and burnout levels. Technological burden is one of the most frequently cited causes of burnout and has been associated with a 29% higher rate of burnout [7, 29, 30]. Few if any studies, however, have investigated the role of population health management tools in alleviating or contributing to high rates of burnout in primary care.

Population health management is a cornerstone of the Veterans’ Health Administration’s (VA) PCMH, which uses team-based structures with specified role delegation (primary care physician, registered nurse, licensed vocational nurse, administrative clerk) to coordinate complex primary care management [31]. In the VA, primary care teams have access to internally developed, EHR-based population health management tools including a risk prediction algorithm,^25^hospitalization and emergency department visit rates, specific medical and mental health diagnoses, quality dashboards and registries, online VA case management software [32], a housing instability indicator, and specific prescription medications. While no studies that we are aware of have compared VA PHMT to those available in other commercial EHR systems, VA’s tools are likely similar in that they share the common goal of improving health outcomes for specific populations. In this study, we investigate the association between clinician ratings of VA EHR-based population health management tools (PHMTs) importance and burnout.

## Methods

### Data source

In this study, we analyzed data from the web-based national VA primary care personnel survey administered between July 16th, 2018 and September 14th, 2018. It assessed demographics, use of access tools, clinic challenges, care management and coordination, work distribution and coordination, staffing, and patient-centeredness. The survey link was emailed directly to all primary care personnel with four email reminder follow-ups to complete the survey. Response was voluntary, and all surveys were anonymous with only clinic identifiers. Respondents included primary care providers (PCPs: physicians, physician assistants, nurse practitioners), registered nurses (RNs), clinical associates (licensed practical nurses, medical assistants), clerical associates, social workers, pharmacists, behavioral health providers, nutritionists, and health educators. For our analytic sample we excluded clerical associates, social workers, pharmacists, behavioral health providers, nutritionists, and health educators because these healthcare workers were less likely to follow patients over time and our preliminary data analysis showed they were less likely to access population health management tools.

### Main measures

The study outcomes include the level and frequency of burnout symptoms. Burnout level was based on a one-item, 5-point measure used for the Physician Worklife Survey, a non-proprietary measure that has been validated and found acceptable as a substitute for the Maslach Burnout Inventory Emotional Exhaustion (MBI:EE) subscale [33, 34]. The item asks: “Overall, based on your definition of burnout, how would you rate your level of burnout?”, with response options 1 = I enjoy my work and have no symptoms of burnout, 2 = Occasionally I am under stress, and I don’t always have as much energy as I once did, but I don’t feel burned out; 3 = I am definitely burning out and have one or more symptoms of burnout, such as physical and emotional exhaustion; 4 = The symptoms of burnout that I’m experiencing won’t go away. I think about frustration at work a lot; and 5 = I feel completely burned out and often wonder if I can go on. I am at the point where I may need some changes or may need to seek some sort of help. For analyses, high burnout level was defined as one or more symptoms = 1 (e.g., response options > 3) and less than 1 symptom = 0 (e.g., response options <  = 2), as has been done in other studies using this measure [33]. Burnout frequency was a single item measure from the MBI:EE (“I feel burned out from my work”) measured on a 7-point scale ranging from never, a few times a year or less, once a month or less, a few times a month, once a week, a few times a week, and every day. It has been validated as a standalone burnout assessment by West and colleagues. For analysis we followed the recommendation of West and colleagues for high burnout frequency defined as once a week or more often = 1 and less than once per week = 0 [30].

Our primary predictors included respondents’ ratings of the importance of 10 population health management tools (3-point Likert scale: very important, somewhat important, not important, with options for don’t use and don’t know). Response options were dichotomized as “Very Important” vs all other responses. These tools included the VA risk prediction algorithm called the Care Assessment Needs score (CAN) [35], hospitalization and emergency department visit rates, specific medical and mental health diagnoses, the Primary Care Almanac (a panel management information tool), the Patient Care Assessment System or PCAS (online VA case management software) [32], local/VISN databases, Opioid Therapy Risk Report (a tool for tracking patients on long-term opioid therapy), External Peer Review Program fallout report (tool to identify patients who are not receiving timely and effective care), housing instability indicator, and specific prescription medications.

Other control variables used in multivariate analyses included: race (non-Hispanic white, Black/African-American, Asian, Spanish/Hispanic/Latinx, other/multirace), gender (male, female, not answered), VA tenure (less than 5 years, 5 years or more), occupation (PCP, RN, LVN), primary care clinic type (medical center-based or community-based outpatient clinic). We also included covariates that prior literature have shown to be predictive of clinician burnout: PCMH team staffed with a ratio of 3.0 team members to each PCP, as specified in the staffing model for VA’s PCMH (yes, no) [36]; changes in staffing (yes, no, don’t know); perception of adequate training for the current role (yes, no); and, aggregated measures of primary care clinical activities (patient care, patient assessment, and response to patient messages) [37].

### Analyses

Bivariate associations of burnout occurrence with demographics were summarized using means with standard deviations or relative frequency, as appropriate. Statistical tests for these differences were assessed using Welch’s t-test or a chi-square test. Multivariable associations of the PHMTs on burnout level and frequency were examined using logistic regression models, conducted separately for each tool and outcome. All models adjusted for the covariates described in the prior section above. Models for burnout frequency were assessed only for the subset who indicated they had experienced any burnout. Sensitivity analyses also examined alternative categorization of the PHMT responses, disaggregating the reference group to have a separate category for “Don’t know/Don’t use” options. Post-hoc analyses examined if the PHMT associations with burnout differed by team role through the use of a PHMT-by-team role interaction term. All analyses accounted for the complex survey design using survey weights and regional strata. Statistical significance was determined based on a two-sided alpha level of 0.05. All analyses were conducted in Stata SE version 16.1, StataCorp LP (College Station, Texas).

The VA Office of Primary Care reviewed the activities reported in this manuscript and determined that this quality improvement effort did not constitute research as described in VHA Office of Research and Development Program Guide 1200. The VA Greater Los Angeles Institutional Review Board conducted an administrative review and concurred that the study activities do not constitute research.

## Results

Our analytic sample size was 4,257 respondents (17.7% response rate). The sample included primarily females (77%), white, non-Hispanic (66%), and consisted of 37% PCPs (medical doctor, nurse practitioner, or physician’s assistant), 38% RNs, and 25% LVNs (Table 1). Forty-one percent of respondents (*n* = 1,828) reported experiencing high burnout and among those, 37% had frequent (at least weekly) burnout symptoms.

**Table 1:** Sample characteristics

		^**a**^**Burnout Occurrence**		
	All *N* = 4,257	No *n* = 2,429	Yes *n* = 1,828	p
^b^Burnout Frequency > 1x/week*, n (% yes)*			1474 (80%)	
Medical center-based primary care clinic*, n (% yes)*	2,178 (52%)	1,220 (51%)	958 (53%)	0.313
Job role, *n (%)*				
^c^Primary care provider	1,531 (37%)	749 (32%)	782 (44%)	< .001
Registered nurse	1,639 (38%)	982 (40%)	657 (35%)	< .001
Licensed practical nurse	1,087 (25%)	698 (28%)	389 (21%)	< .001
Delegation of clinical duties, *(n) %* ± *SD*				
Patient care	(4,257) 14.9% ± 4.6%	(2,429) 15.1% ± 4.6%	(1,828) 14.7% ± 4.7%	0.012
Assessing patients	(4,257) 11.6% ± 4.5%	(2,429) 11.9% ± 4.4%	(1,828) 11.2% ± 4.5%	< .001
Responding to messages	(4,257) 8.27% ± 2.96%	(2,429) 8.21% ± 2.95%	(1,828) 8.34% ± 2.97%	0.241
Team staffed at 3:1 provider ratio, *n (% yes)*	2,558 (64%)	1,598 (70%)	960 (55%)	< .001
Team staffing changes/losses in past year,* n (% yes)*	2,779 (67%)	1,508 (64%)	1,271 (71%)	< .001
Perception of adequate training to “function at the top of my scope of practice”, *n (% yes)*	3,026 (72%)	1,989 (83%)	1,037 (58%)	< .001
Worked for VA for 5 or more years, *n (% yes)*	2,480 (61%)	1,317 (57%)	1,163 (66%)	< .001
Race/ethnicity, *n (%)*				0.008
Non-Hispanic white	2,763 (66%)	1,574 (65%)	1,189 (67%)	
Non-Hispanic black	272 (8%)	184 (9%)	88 (6%)	
Hispanic	316 (8%)	171 (8%)	145 (9%)	
Non-Hispanic Asian	359 (10%)	219 (11%)	140 (9%)	
Other	282 (7%)	146 (6%)	136 (8%)	
Sex, *n (%)*				0.009
Male	907 (23%)	478 (21%)	429 (25%)	
Female	3,090 (77%)	1,819 (79%)	1,271 (75%)	

^a^Burnout occurrence defined by the presence of 1 or more burnout symptoms

^b^Burnout frequency defined as once a week or more among those with burnout

^c^Defined as a medical doctor, nurse practitioner, or physician’s assistant

Table 2 shows the bivariate analyses of PHMT ratings and burnout level and frequency. A large proportion (range of 7–55%) of respondents indicated that they don’t know or don’t use PHMT tools. Among those that evaluated the tools importance, the majority of responders indicated that they believed PHMTs to be either “Somewhat important” or “Very important”, with the largest category indicating “Very important”. The bivariates additionally showed consistent patterns with higher ratings of PHMT importance associated with lower burnout level and frequency (Table 2).

**Table 2:** Percent experiencing burnout by population health management tools

Population Health Management Tool (PHMT)	PHMT Distribution	^a^High Burnout Level	^b^High Burnout Frequency
Care Assessment Need (CAN) score			
Don’t know/Don’t use	1,080 (25%)	47%	41%
Not important	248 (6%)	64%	61%
Somewhat important	1,150 (27%)	45%	42%
Very important	1,806 (42%)	36%	32%
Recent hospitalizations and ED visits			
Don’t know/Don’t use	312 (7%)	50%	44%
Not important	62 (1%)	58%	60%
Somewhat important	669 (16%)	51%	46%
Very important	3,241 (76%)	40%	36%
Specific medical and mental health diagnoses			
Don’t know/Don’t use	641 (15%)	48%	42%
Not important	86 (2%)	58%	58%
Somewhat important	928 (22%)	47%	42%
Very important	2,629 (61%)	40%	36%
Primary Care Almanac			
Don’t know/Don’t use	1,129 (26%)	44%	39%
Not important	171 (4%)	68%	64%
Somewhat important	984 (23%)	49%	45%
Very important	2,000 (47%)	38%	33%
Patient Care Assessment System (PCAS)			
Don’t know/Don’t use	2,415 (55%)	46%	41%
Not important	172 (4%)	67%	64%
Somewhat important	614 (14%)	41%	37%
Very important	1,083 (26%)	34%	31%
Local/VISN Database			
Don’t know/Don’t use	1,853 (43%)	47%	42%
Not important	159 (4%)	62%	62%
Somewhat important	786 (18%)	44%	40%
Very important	1,486 (35%)	35%	31%
Opioid Risk Report(OTRR)			
Don’t know/Don’t use	1,436 (34%)	45%	41%
Not important	157 (4%)	60%	59%
Somewhat important	868 (20%)	48%	44%
Very important	1,823 (43%)	37%	33%
Quality Metric Fallout Reports			
Don’t know/Don’t use	2,220 (52%)	45%	41%
Not important	277 (7%)	59%	57%
Somewhat important	804 (19%)	42%	38%
Very important	983 (23%)	34%	29%
Housing Instability			
Don’t know/Don’t use	2,140 (50%)	44%	40%
Not important	194 (5%)	55%	52%
Somewhat important	879 (21%)	45%	39%
Very important	1,071 (25%)	37%	33%
Specific Prescription Medications			
Don’t know/Don’t use	1,133 (26%)	46%	42%
Not important	87 (2%)	48%	47%
Somewhat important	848 (20%)	48%	42%
Very important	2,216 (52%)	39%	35%

^a^High burnout level defined by the presence of 1 or more burnout symptoms. Values reported within PHMT categories

^b^High burnout frequency defined as once a week or more among those with burnout. Values reported within PHMT categories

For eight of the 10 PHMT, multivariate analyses (Table 3) showed lower odds of high burnout level associated with high PHMT ratings, indicating 15% to 30% lower odds among those who indicated the PHMT was “Very Important” as compared with a rating of “Somewhat/Not/Don’t know/Don’t use”. The lowest odds of high burnout were for the ratings of local registry/database with an odds ratio (OR) of 0.70 (95% CI: 0.59, 0.82, *p* < 0.001). Of the ten PHMTs examined, only the ratings for housing instability and specific prescription medications, were not significantly associated with high burnout (OR = 0.84, *p* > .05 and OR = 0.85, *p* > .05, respectively). Similarly, a “very important” rating was associated with lower odds of frequent burnout for eight of the 10 PHMT, ranging from 12–36% lower.

**Table 3:** Odds ratios of PHMT “Very Important” rating as a predictor of high burnout level and frequency

	^**a**^**High Burnout Level** *Any Symptoms vs None*		^**b**^**High Burnout Frequency** *Once a week or more vs less*	
	**OR (95% CI)**	**p**	**OR (95% CI)**	**p**
Care Assessment Need (CAN) score				
* DK/NR/Not Important/Somewhat Important*	Ref		Ref	
* Very Important*	0.73 (0.61, 0.87)	< .001	0.71 (0.60, 0.84)	< .001
Recent hospitalizations and ED visits				
* DK/NR/Not Important/Somewhat Important*	Ref		Ref	
* Very Important*	0.73 (0.61, 0.88)	< .001	0.74 (0.61, .89)	< .01
Specific medical and mental health diagnoses				
* DK/NR/Not Important/Somewhat Important*	Ref		Ref	
* Very Important*	0.83 (0.72, 0.97)	< .05	0.88 (0.76, 1.02)	ns
Primary Care Almanac				
* DK/NR/Not Important/Somewhat Important*	Ref		Ref	
* Very Important*	0.84 (0.68, 0.93)	< .01	0.77 (0.66, 0.90)	< .01
Patient Care Assessment System (PCAS)				
* DK/NR/Not Important/Somewhat Important*	Ref		Ref	
* Very Important*	0.73 (0.61, 0.88)	< .001	0.77 (0.64, 0.93)	< .01
Local/VISN Database				
* DK/NR/Not Important/Somewhat Important*	Ref		Ref	
* Very Important*	0.70 (0.59, 0.82)	< .001	0.68 (0.58, 0.81)	< .001
Opioid Risk Report(OTRR)				
* DK/NR/Not Important/Somewhat Important*	Ref		Ref	
* Very Important*	0.76 (0.64, 0.90)	< .01	0.68 (0.58, 0.81)	< .001
Quality Metric Fallout Reports				
* DK/NR/Not Important/Somewhat Important*	Ref		Ref	
* Very Important*	0.75 (0.61, 0.92)	< .01	0.64 (0.52, 0.80)	< .001
Housing Instability				
* DK/NR/Not Important/Somewhat Important*	Ref		Ref	
* Very Important*	0.84 (0.70, 1.03)	ns	0.80 (0.66, 0.97)	< .05
Specific Prescription Medications				
* DK/NR/Not Important/Somewhat Important*	Ref		Ref	
* Very Important*	0.85 (0.73, 1.03)	ns	0.85 (0.72, 1.01)	ns

Models adjusted for sex, race/ethnicity, medical center-based primary care clinic or community based primary care clinic, delegation of clinical duties, staffing at 3:1 provider ratios, team staffing changes/losses in past year, having worked for VA for 5 or more years, and having received adequate training

*DK* Don’t Know, *NR* No Response, *OR* Odds Ratio, *CI* Confidence Interval, *Ref* Reference

^a^High Burnout level defined by the presence of 1 or more burnout symptoms

^b^High Burnout frequency defined as once a week or more among those with burnout

Sensitivity analyses were additionally conducted to ensure comparisons of “Very Important” to “Somewhat/Not Important” were similar when excluding the “don’t know/don’t use” responses. Conclusions were similar showing 6 of 10 PHMTs associated with high burnout level and 3 of 10 with high burnout frequency (supplementary Table S1). Lastly, we examined tests for differential effects of the PHMT associations on burnout by team role (e.g. primary care provider, RN, and LPN). These tests for interaction were not statistically significant (supplementary Table S2).

## Discussion

This study investigated the relationship between burnout and the perceived importance of EHR-based clinical tools that are available to PCMH teams in all VA primary care clinics nationally. Although burnout levels and drivers for all healthcare disciplines have been well documented in the literature [38], and current research had begun to focus on interventions to address burnout [39], this is the first study we are aware of that evaluates the association of population health management tools with clinician burnout level and frequency among a large national sample of primary care providers and nurses. This study demonstrated that perceived importance of population health management tools was associated with lower likelihoods of high clinician burnout, even after controlling for other known drivers of burnout such as staffing and individual demographic characteristics.

Many studies conducted in VA and non-VA settings have shown that workplace factors (e.g., workload, understaffing, work/life balance, job autonomy, and perceived leadership support) have strong associations with burnout, absenteeism, productivity, and turnover [38, 40, 41]. Evidence from recent systematic reviews of interventions to address burnout and psychological well-being among HCWs support effectiveness of organization-directed (e.g., reduced workload, flexible work schedules, redesigning workflows, quality improvement) and individual-directed (e.g., mindfulness-based stress reduction, meditation, communication skills-training) interventions, with larger effect sizes attributed to the former [4]. One recent review of 282 workplace interventions aimed at reducing or preventing burnout described the evidence base as poor [39]. Our study has important implications for healthcare administrators considering organizational interventions to address burnout that harness information technology to streamline or reduce workload.

Although we found significant associations between higher ratings on PHMT and both burnout outcomes (level and frequency), we could not establish causality or the direction of the association between burnout and tool ratings. One possible explanation for our findings is that population health management tools may be protective against clinician burnout. PHMTs are intended to streamline care and implement preventative medicine measures by allowing practitioners to identify high-risk patients and intervene on their needs before the condition progresses or medical complications arise [42]. For instance, recognition of persistently elevated blood pressures on a panel management information tool may compel physicians to bring in that subset of patients for more frequent visits, recommend exercise services offered by the VA, or refer them to a nutritionist to optimize their diet. These interventions may prevent subsequent comorbidities that then require more intensive management with medication titration, subspecialty referrals, and frequent monitoring. Preventative interventions have been repeatedly demonstrated to reduce patient morbidity, mortality [43], and hospital costs [44], and medical complexity in patient cohorts has been linked to clinician burnout [42]. By preventing medical complexity through guideline-based preventive services, population health management tools may in turn prevent clinician burnout. Another mechanism by which PHMTs may reduce provider burnout is by streamlining primary care workflow [45]. In particular, a hospitalization and ED visits tracker can alert providers to patients who require post-hospitalization or ED follow-up visits, rather than having providers search through each patient’s chart individually for recent occurrences. If PHMTs do reduce provider burnout by preventing medical complexity and streamlining workflow, increased efforts should be made to integrate PHMT use into the PCMH clinical workflow and create greater reward systems to ensure compliance.

Conversely, the burden of population health management may precipitate clinician burnout by increasing IT and overall task burden. The majority of the VA PHMTs require increased EHR clicks and accessing additional portals that house external patient dashboards, both of which result in increased screen time and clinical task burden [7, 29, 30]. They may prompt providers to perform more patient care tasks such as ordering more tests and studies, arranging follow up visits, changing medical prescriptions, and/or referring to subspecialty providers. It is also possible that providers who report high burnout are less likely to use population health management tools, potentially due to lack of physical or emotional bandwidth, lack of motivation or engagement, or psychiatric conditions precipitated by burnout [46]. In fact, prior studies have demonstrated that burned-out clinicians are less able to accurately evaluate their clinic’s available social resources, so they may perceive the clinic to have low efficacy to address social needs even when the clinic is fully equipped to do so [47]. Burned out providers may not be motivated or aware of clinic resources to intervene on patient care, much less take a proactive approach in identifying high-risk patients through PHMTs.

Increasingly, novel applications of artificial intelligence and other virtual technologies are entering clinical workflows to further enhance PHMTs. Clinically-oriented customer relationship management (CRM) software and clinical intelligence platforms such as Salesforce’s Health Cloud [48], Clint Health [49], and Alvee Health [50] ingest large population health databases to discern care gaps and generate actionable insights. Patient engagement platforms such as Healow [51] are already used in multiple practice sites and geographies. Scribing technology augmented by machine learning and large language models such as Nuance [52], Deep Scribe [53], and Abridge [54] hold the potential to streamline documentation and are being deployed in numerous health systems. In conjunction with existing PHMTs, these new technologies have the potential to expand the capabilities of population health management to improve patient outcomes as well as reduce clinician burnout. Further research is needed, however, to investigate the rapidly evolving application of virtual technologies, including the potential benefits and unintended consequences for healthcare systems, providers, and patients and their families.

This study had several limitations, with the most important of these being the cross-sectional nature of the survey data. As stated above, we were not able to determine a causal relationship between burnout and tool ratings. Second, we used single-item measures of burnout which, although validated against more comprehensive multi-item measures, may not have captured the full range of domains encompassed by this multi-faceted phenomenon. Third, the VA is a national integrated healthcare system with standardized care protocols and team structures that may not be applicable to the diversity of health care delivery practices in the United States. The VA PHMTs that were the subject of this study were internally developed and may not be directly comparable to those embedded in other commercial EHR systems; we were not able in this study to compare VA PHMTs to others available commercially. Also, our results are subject to biases inherent in survey research, including non-response bias. Although the sample size was large, the response rate was low, and thus our results should be interpreted with caution and may not be generalizable across national healthcare systems. Finally, the set of sampled population health management tools is not all-encompassing. For example, clinical “reminders” embedded within each primary care visit (anxiety, depression, tobacco use, cancer, and vaccine screening) were not assessed by this survey. The function of these reminders aligns with those of PHMTs, and their inclusion in the survey may have elucidated further insights on clinician burnout because they are used in every primary care encounter.

Nevertheless, our study has important implications for healthcare leaders and decision makers. It is important to determine whether population health management tools play a role in mitigating or perpetuating provider burnout. Burned-out clinicians may not consider the EHR-based tools important and may not be using them to perform care management. Although this study could not determine why burned-out clinicians may not find the tools useful, if the tools create additional technological burden, they may need adaptation to make them more accessible, more intuitive, and less burdensome to use. Likewise, if use of the tools streamlines the work of population health management and/or results in less workload due to patients with poorly managed chronic conditions, finding ways to improve tool use may also alleviate burnout, which has become a national crisis [55]. Regardless, future studies using longitudinal data are necessary to determine whether burnout results in less interest/use of tools for population health management, or whether the tools may be protective against burnout. Qualitative studies are also needed to understand further the relationship between population health management more broadly and burnout, and whether the design of tools could be improved to reduce the technology burden associated with their use.

## Conclusion

In conclusion, our study revealed a strong association between high ratings on EHR-based VA standardized population health management tools and low primary care provider burnout. More research is needed to determine the causal direction of this association, including qualitative interviews with clinicians to understand the causal mechanisms and longitudinal surveys with large, national samples. Regardless of the causal direction of this association, our study suggests that healthcare administrators should carefully consider the potential workplace impacts of introducing population health management tools for healthcare clinicians and trainees and the potential for unintended increased workload such tools may entail. To provide healthcare administrators and decision makers with information to improve the usefulness of EHR-based population health management tools, future studies will need to establish the existence and direction of a causal relationship between burnout and tool use, and identify the reasons why clinicians do not find these tools important for performing population health management.

### Supplementary Information

**Supplementary Material 1. **

## Data Availability

The data that support the findings of this study are available from the Primary Care Analytics Team within the VHA Office of Primary Care, but restrictions apply to the availability of these data, which were used under a Designation of Non-Research Activity (DNRA) for the current study, and so are not publicly available. Data are however available from the authors upon reasonable request and with permission of the VHA Office of Primary Care.
