# Supplementary material for: The association between population health management tools and clinician burnout in the United States VA primary care patient-centered medical home
Source: BMC Prim Care. 2024 May 15;25:164. doi: 10.1186/s12875-024-02410-8 (PMC11094957; doi:10.1186/s12875-024-02410-8)
Supplement: Supplementary file 1 — Supplementary Material 1. [file 12875_2024_2410_MOESM1_ESM.docx]

| Supplementary Table S1– Multicategorical PHMT Predictors | | | | |
| --- | --- | --- | --- | --- |
|  | **^1^High Burnout Level**  *Any Symptoms vs None* | | **^2^High Burnout Frequency**  *Once a week or more vs less* | |
|  | **OR (95% CI)** | **p** | **OR (95% CI)** | **p** |
| Care Assessment Need (CAN) score |  |  |  |  |
| *Not Important/Somewhat Important* | Ref |  | Ref |  |
| *Don’t Know/No Response* | 0.99 (0.80, 1.22) | 0.917 | 0.91 (0.60, 1.37) | 0.644 |
| *Very Important* | 0.72 (0.59, 0.87) | <.001 | 0.68 (0.47, 1.00) | 0.050 |
| Recent hospitalizations and ED visits |  |  |  |  |
| *Not Important/Somewhat Important* | Ref |  | Ref |  |
| *Don’t Know/No Response* | 1.11 (0.79, 1.58) | 0.543 | 1.42 (0.75, 2.70) | 0.280 |
| *Very Important* | 0.76 (0.62, 0.93) | 0.008 | 0.96 (0.64, 1.45) | 0.857 |
| Specific medical and mental health diagnoses |  |  |  |  |
| *Not Important/Somewhat Important* | Ref |  | Ref |  |
| *Don’t Know/No Response* | 1.17 (0.91, 1.51) | 0.224 | 1.26 (0.79, 2.02) | 0.336 |
| *Very Important* | 0.90 (0.76, 1.07) | 0.218 | 1.13 (0.81, 1.58) | 0.479 |
| Primary Care Almanac |  |  |  |  |
| *Not Important/Somewhat Important* | Ref |  | Ref |  |
| *Don’t Know/No Response* | 0.81 (0.66, 0.99) | 0.042 | 0.85 (0.56, 1.28) | 0.433 |
| *Very Important* | 0.72 (0.60, 0.86) | <.001 | 0.62 (0.44, 0.87) | 0.005 |
| Patient Care Assessment System (PCAS) |  |  |  |  |
| *Not Important/Somewhat Important* | Ref |  | Ref |  |
| *Don’t Know/No Response* | 0.98 (0.79, 1.20) | 0.819 | 0.95 (0.65, 1.40) | 0.800 |
| *Very Important* | 0.71 (0.56, 0.91) | 0.005 | 0.87 (0.56, 1.35) | 0.523 |
| Local/VISN Database |  |  |  |  |
| *Not Important/Somewhat Important* | Ref |  | Ref |  |
| *Don’t Know/No Response* | 1.05 (0.87, 1.28) | 0.588 | 1.03 (0.71, 1.49) | 0.862 |
| *Very Important* | 0.71 (0.57, 0.87) | 0.001 | 0.78 (0.52, 1.16) | 0.211 |
| Opioid Risk Report (OTRR) |  |  |  |  |
| *Not Important/Somewhat Important* | Ref |  | Ref |  |
| *Don’t Know/No Response* | 1.07 (0.87, 1.31) | 0.527 | 0.91 (0.59, 1.40) | 0.665 |
| *Very Important* | 0.81 (0.66, 0.99) | 0.036 | 0.60 (0.41, 0.87) | 0.006 |
| Quality Metric Fallout Reports |  |  |  |  |
| *Not Important/Somewhat Important* | Ref |  | Ref |  |
| *Don’t Know/No Response* | 1.17 (0.97, 1.41) | 0.109 | 1.11 (0.79, 1.55) | 0.561 |
| *Very Important* | 0.85 (0.66, 1.09) | 0.202 | 0.70 (0.47, 1.04) | 0.075 |
| Housing Instability |  |  |  |  |
| *Not Important/Somewhat Important* | Ref |  | Ref |  |
| *Don’t Know/No Response* | 1.07 (0.89, 1.30) | 0.466 | 1.41 (1.02, 1.94) | 0.037 |
| *Very Important* | 0.87 (0.70, 1.08) | 0.216 | 1.03 (0.69, 1.55) | 0.872 |
| Specific Prescription Medications |  |  |  |  |
| *Not Important/Somewhat Important* | Ref |  | Ref |  |
| *Don’t Know/No Response* | 1.20 (0.98, 1.46) | 0.078 | 1.25 (0.83, 1.88) | 0.293 |
| *Very Important* | 0.95 (0.78, 1.15) | 0.600 | 1.02 (0.71, 1.45) | 0.923 |
| ^1^High Burnout level defined by the presence of 1 or more burnout symptoms ^2^High Burnout frequency defined as once a week or more among those with burnout  OR=Odds Ratio; CI=Confidence Interval; Ref=Reference  Models adjusted for sex, race/ethnicity, Medical center-based primary care clinic, delegation of clinical duties, staffing at 3:1 provider ratios, team staffing changes/losses in past year, having worked for VA for 5 or more years, and having received adequate training. | | | | |

| Supplementary Table S2 –Interactions with Team Role | | | | |
| --- | --- | --- | --- | --- |
|  | **^1^High Burnout Level** | | **^2^High Burnout Frequency** | |
| *Interaction with Team Role -* | *Binary Predictor Interaction p* | *Multicategorical Predictor Interaction p* | *Binary Predictor Interaction p* | *Multicategorical Predictor Interaction p* |
| Care Assessment Need (CAN) score | 0.370 | 0.743 | 0.350 | 0.906 |
| Recent hospitalizations and ED visits | 0.262 | 0.462 | 0.177 | 0.584 |
| Specific medical and mental health diagnoses | 0.124 | 0.381 | 0.176 | 0.641 |
| Primary Care Almanac | 0.371 | 0.467 | 0.459 | 0.846 |
| Patient Care Assessment System (PCAS) | 0.882 | 0.028 | 0.861 | 0.098 |
| Local/VISN Database | 0.357 | 0.506 | 0.536 | 0.694 |
| Opioid Risk Report (OTRR) | 0.898 | 0.544 | 0.694 | 0.398 |
| Quality Metric Fallout Reports | 0.454 | 0.705 | 0.404 | 0.833 |
| Housing Instability | 0.371 | 0.601 | 0.205 | 0.614 |
| Specific Prescription Medications | 0.776 | 0.938 | 0.969 | 0.840 |
| ^1^High Burnout level defined by the presence of 1 or more burnout symptoms ^2^High Burnout frequency defined as once a week or more among those with burnout  DK=Don’t Know; NR=No Response; OR=Odds Ratio; CI=Confidence Interval; Ref=Reference  Models adjusted for sex, race/ethnicity, Medical center-based primary care clinic, delegation of clinical duties, staffing at 3:1 provider ratios, team staffing changes/losses in past year, having worked for VA for 5 or more years, and having received adequate training. | | | | |
